# *N*,*N*′-Bis[(2-hydroxy­phen­yl)(phen­yl)methyl­idene]propane-1,2-diamine

**DOI:** 10.1107/S1600536810015291

**Published:** 2010-05-08

**Authors:** Robert S. Black, David G. Billing, Agata Bartyzel, Ewa M. Cukrowska

**Affiliations:** a Molecular Sciences Institute, School of Chemistry, University of the Witwatersrand, Private Bag 3, PO Wits, 2050, South Africa

## Abstract

In the the title compound, C_29_H_26_N_2_O_2_, two strong intra­molecular O—H⋯N hydrogen bonds involving the hydr­oxy and imine groups generate *S*(6) ring motifs. The dihedral angles between the pairs of terminal benzene rings are 89.8 (2) and 87.8 (2)°.

## Related literature

For related compounds and further synthetic details, see: Schilf *et al.* (2007 ▶). For intra­molecular hydrogen bonds in this type of compound, see: Fernández-G *et al.* (2001 ▶); Kabak (2003 ▶); Wojciechowski *et al.* (2001 ▶); Dey *et al. (*2001); Koşar *et al.* (2004 ▶); Lu *et al.* (2008 ▶); Qiu & Zhao (2008 ▶); Montazerozohori *et al.* (2009 ▶); Corden *et al.* (1996 ▶); Black *et al.* (2010 ▶); Dey *et al.* (2001 ▶).
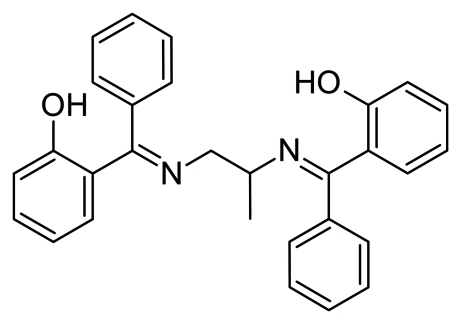


## Experimental

### 

#### Crystal data

C_29_H_26_N_2_O_2_
*M* *_r_* = 434.52
Monoclinic, 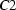

*a* = 18.1766 (8) Å
*b* = 7.9808 (4) Å
*c* = 16.0347 (8) Å
β = 92.703 (2)°
*V* = 2323.47 (19) Å^3^
*Z* = 4
Mo *K*α radiation
μ = 0.08 mm^−1^
*T* = 296 K
0.62 × 0.38 × 0.24 mm

#### Data collection

Bruker APEXII CCD diffractometer
Absorption correction: integration (*XPREP*; Bruker, 1999 ▶) *T*_min_ = 0.918, *T*_max_ = 1.000
22291 measured reflections
3001 independent reflections
2724 reflections with *I* > 2σ(*I*)
*R*_int_ = 0.026

#### Refinement

*R*[*F* ^2^ > 2σ(*F* ^2^)] = 0.034
*wR*(*F* ^2^) = 0.087
*S* = 1.05
3001 reflections
301 parameters
1 restraint
H-atom parameters constrained
Δρ_max_ = 0.20 e Å^−3^
Δρ_min_ = −0.18 e Å^−3^

### 

Data collection: *APEX2* (Bruker, 2005 ▶); cell refinement: *SAINT-NT* (Bruker, 2005 ▶); data reduction: *SAINT-NT*; program(s) used to solve structure: *SHELXTL* (Sheldrick, 2008 ▶); program(s) used to refine structure: *SHELXL97* (Sheldrick, 2008 ▶); molecular graphics: *PLATON* (Spek, 2009 ▶); software used to prepare material for publication: *WinGX* (Farrugia, 1999 ▶) and *PLATON*.

## Supplementary Material

Crystal structure: contains datablocks global, I. DOI: 10.1107/S1600536810015291/hb5392sup1.cif

Structure factors: contains datablocks I. DOI: 10.1107/S1600536810015291/hb5392Isup2.hkl

Additional supplementary materials:  crystallographic information; 3D view; checkCIF report

## Figures and Tables

**Table 1:** Selected torsion angles (°)

C1—C6—C7—C8	89.8 (2)
C17—C22—C23—C24	87.8 (2)

**Table 2:** Hydrogen-bond geometry (Å, °)

*D*—H⋯*A*	*D*—H	H⋯*A*	*D*⋯*A*	*D*—H⋯*A*
O1—H1*A*⋯N1	0.82	1.84	2.573 (2)	147
O2—H2*A*⋯N2	0.82	1.83	2.553 (2)	147

**Fig. 1.**
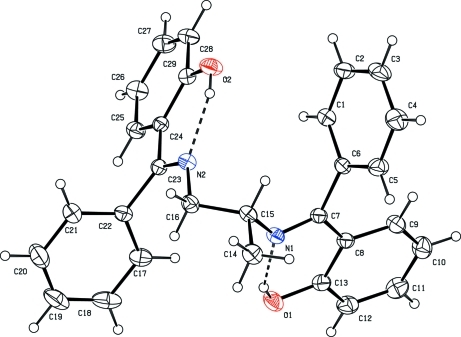
The molecular structure of (I) drawn at the 30% probability displacement ellipsoids. Hydrogens bonds are shown as dashed lines.

## supplementary crystallographic information

### Comment

The molecular structure of the title compound form two strong intermolecular hydrogen bonds O—H···N involving the hydroxyl and the imine groups, forming S(6) ring motifs which are common to this type of compound (Schilf *et al.*, 2007) and (Fernández-G *et al.*, 2001), and also seen in the work completed by (Kabak *et al.*, 2003), (Wojciechowski *et al.*, 2001), (Dey *et al.*, 2001), (Koşar, *et al.*, 2004), (Lu, *et al.*, 2008) (Qiu & Zhao, 2008), (Montazerozohori *et al.*, 2009), (Corden *et al.*, 1996) and (Black *et al.*, 2010). This causes the dihedral angles between the adjacent phenyl rings and phenyl containing plains to be (C1—C6—C7—C8) 89.8 (2)° and (C17—C22—C23—C24) 87.8 (2)° respectively. These dihedral angles are comparable to (Corden *et al.*, 1996) and (Black *et al.*, 2010). The stereogenic centre on the methyl substituted carbon C15 allows the system to pack in the noncentrosymmetric space group *C2*. The remaining weak interactions in the crystals form unexceptional σ-π interactions.

### Experimental

A mixture of 0.01 mol (2.00 g) of 2-hydroxybenzophenone and 0.005 mole (0.42 ml) of 1,2-propanediamine in 40 ml of methanol was refluxed for 7 h. The excess of solvent (ca. 30 ml) was then evaporated. After cooling to 277 K, a yellow solid was produced. The polycrystalline product was collected by filtration, washed with methanol and dried a yield 54% was obtained. Recrystalization from an ethanol solution yielded yellow blocks of (I). Elemental analysis: *C% 79.67 H% 5.99 N% 6.03*.

### Refinement

The absolute structure of (I) is indeterminate based on the present refinement. All H atoms were refined using a riding model, with a C—H distance of 0.96, for Ar—H a distance of 0.93 Å and for O—H a distance of 0.82 Å, and *U*iso(H) = 1.2*U*eq(C) and 1.5*U*eq(O). The highest residual peak was 0.742 Å from atom C6 with a ρ = 0.20 e Å^-3^.

### Crystal data

**Table:**

C_29_H_26_N_2_O_2_	*F*(000) = 920
*M**_r_* = 434.52	*D*_x_ = 1.242 Mg m^−^^3^
Monoclinic, *C*2	Mo *K*α radiation, λ = 0.71073 Å
Hall symbol: C 2y	Cell parameters from 9931 reflections
*a* = 18.1766 (8) Å	θ = 2.2–28.3°
*b* = 7.9808 (4) Å	µ = 0.08 mm^−^^1^
*c* = 16.0347 (8) Å	*T* = 296 K
β = 92.703 (2)°	Block, yellow
*V* = 2323.47 (19) Å^3^	0.62 × 0.38 × 0.24 mm
*Z* = 4	

### Data collection

**Table:**

Bruker APEXII CCD diffractometer	3001 independent reflections
Radiation source: sealed tube	2724 reflections with *I* > 2σ(*I*)
graphite	*R*_int_ = 0.026
φ and ω scans	θ_max_ = 28°, θ_min_ = 1.3°
Absorption correction: integration (*XPREP*; Bruker, 1999)	*h* = −23→24
*T*_min_ = 0.918, *T*_max_ = 1.000	*k* = −10→10
22291 measured reflections	*l* = −21→20

### Refinement

**Table:**

Refinement on *F*^2^	1 restraint
Least-squares matrix: full	H-atom parameters constrained
*R*[*F*^2^ > 2σ(*F*^2^)] = 0.034	*w* = 1/[σ^2^(*F*_o_^2^) + (0.0448*P*)^2^ + 0.6417*P*] where *P* = (*F*_o_^2^ + 2*F*_c_^2^)/3
*wR*(*F*^2^) = 0.087	(Δ/σ)_max_ = 0.001
*S* = 1.05	Δρ_max_ = 0.20 e Å^−^^3^
3001 reflections	Δρ_min_ = −0.18 e Å^−^^3^
301 parameters	

### Special details

**Table:**

Experimental. Numerical integration absorption corrections based on indexed crystal faces were applied using the XPREP routine (Bruker, 1999)
Geometry. All esds (except the esd in the dihedral angle between two l.s. planes) are estimated using the full covariance matrix. The cell esds are taken into account individually in the estimation of esds in distances, angles and torsion angles; correlations between esds in cell parameters are only used when they are defined by crystal symmetry. An approximate (isotropic) treatment of cell esds is used for estimating esds involving l.s. planes.

### Fractional atomic coordinates and isotropic or equivalent isotropic displacement parameters (Å^2^)

**Table:**

	*x*	*y*	*z*	*U*_iso_*/*U*_eq_	
C1	0.17117 (10)	0.8773 (3)	0.19104 (12)	0.0380 (4)	
H1	0.1576	0.8561	0.2453	0.046*	
C2	0.23795 (11)	0.9528 (3)	0.17780 (14)	0.0452 (5)	
H2	0.2699	0.9792	0.2229	0.054*	
C3	0.25725 (11)	0.9890 (3)	0.09795 (15)	0.0500 (5)	
H3	0.302	1.0411	0.0891	0.06*	
C4	0.21054 (12)	0.9484 (3)	0.03135 (14)	0.0536 (5)	
H4	0.2237	0.974	−0.0225	0.064*	
C5	0.14368 (11)	0.8693 (3)	0.04351 (12)	0.0432 (5)	
H5	0.1124	0.8412	−0.0019	0.052*	
C6	0.12418 (9)	0.8328 (2)	0.12419 (11)	0.0318 (4)	
C7	0.05455 (9)	0.7387 (2)	0.14143 (10)	0.0308 (4)	
C8	−0.01204 (10)	0.8365 (2)	0.15614 (11)	0.0317 (4)	
C9	−0.01477 (11)	1.0091 (2)	0.14096 (13)	0.0398 (4)	
H9	0.0267	1.0624	0.1219	0.048*	
C10	−0.07704 (12)	1.1025 (3)	0.15342 (13)	0.0478 (5)	
H10	−0.0777	1.2167	0.142	0.057*	
C11	−0.13872 (12)	1.0242 (3)	0.18322 (14)	0.0487 (5)	
H11	−0.1808	1.0867	0.1924	0.058*	
C12	−0.13812 (11)	0.8549 (3)	0.19929 (13)	0.0445 (5)	
H12	−0.1796	0.8042	0.2199	0.053*	
C13	−0.07625 (10)	0.7587 (3)	0.18505 (12)	0.0372 (4)	
C14	0.09851 (12)	0.3771 (3)	0.04854 (12)	0.0445 (5)	
H14A	0.0548	0.3114	0.0545	0.067*	
H14B	0.139	0.3044	0.0374	0.067*	
H14C	0.0907	0.4544	0.0031	0.067*	
C15	0.11599 (10)	0.4740 (2)	0.12901 (11)	0.0330 (4)	
H15	0.1592	0.5453	0.1221	0.04*	
C16	0.13115 (11)	0.3541 (2)	0.20186 (11)	0.0364 (4)	
H16A	0.0869	0.2909	0.2124	0.044*	
H16B	0.1695	0.2755	0.1882	0.044*	
C17	−0.00368 (11)	0.3145 (3)	0.35288 (13)	0.0447 (5)	
H17	−0.0215	0.4205	0.3389	0.054*	
C18	−0.05238 (12)	0.1829 (4)	0.36563 (14)	0.0602 (7)	
H18	−0.1029	0.2012	0.3604	0.072*	
C19	−0.02579 (16)	0.0266 (4)	0.38587 (16)	0.0640 (7)	
H19	−0.0585	−0.0607	0.3944	0.077*	
C20	0.04851 (16)	−0.0019 (3)	0.39361 (17)	0.0639 (7)	
H20	0.066	−0.1085	0.4071	0.077*	
C21	0.09762 (13)	0.1270 (3)	0.38147 (14)	0.0477 (5)	
H21	0.148	0.1073	0.3869	0.057*	
C22	0.07149 (10)	0.2860 (2)	0.36114 (11)	0.0331 (4)	
C23	0.12560 (9)	0.4238 (2)	0.34679 (11)	0.0307 (3)	
C24	0.14855 (9)	0.5331 (2)	0.41770 (11)	0.0304 (3)	
C25	0.11331 (10)	0.5252 (2)	0.49331 (12)	0.0368 (4)	
H25	0.0749	0.4495	0.4988	0.044*	
C26	0.13414 (11)	0.6270 (3)	0.56007 (13)	0.0437 (5)	
H26	0.1102	0.6195	0.6099	0.052*	
C27	0.19109 (12)	0.7405 (3)	0.55188 (14)	0.0477 (5)	
H27	0.2049	0.8105	0.5963	0.057*	
C28	0.22735 (11)	0.7505 (3)	0.47865 (13)	0.0461 (5)	
H28	0.2658	0.8264	0.4741	0.055*	
C29	0.20693 (9)	0.6482 (2)	0.41146 (11)	0.0355 (4)	
N1	0.05252 (8)	0.57835 (19)	0.14636 (9)	0.0338 (3)	
N2	0.15442 (8)	0.44924 (19)	0.27629 (9)	0.0348 (3)	
O1	−0.07848 (8)	0.59298 (19)	0.20016 (11)	0.0509 (4)	
H1A	−0.0408	0.5489	0.1844	0.076*	
O2	0.24508 (8)	0.6613 (2)	0.34177 (8)	0.0487 (4)	
H2A	0.2264	0.6006	0.3053	0.073*	

### Atomic displacement parameters (Å^2^)

**Table:**

	*U*^11^	*U*^22^	*U*^33^	*U*^12^	*U*^13^	*U*^23^
C1	0.0384 (10)	0.0346 (9)	0.0407 (9)	−0.0049 (8)	0.0003 (8)	−0.0001 (8)
C2	0.0365 (10)	0.0390 (10)	0.0591 (12)	−0.0070 (8)	−0.0073 (8)	−0.0052 (9)
C3	0.0296 (10)	0.0468 (12)	0.0742 (14)	−0.0096 (9)	0.0104 (9)	−0.0005 (11)
C4	0.0464 (11)	0.0669 (14)	0.0487 (11)	−0.0111 (11)	0.0144 (9)	0.0084 (11)
C5	0.0386 (10)	0.0536 (12)	0.0374 (9)	−0.0087 (9)	0.0009 (8)	0.0026 (9)
C6	0.0301 (9)	0.0270 (8)	0.0383 (9)	−0.0033 (7)	0.0021 (7)	0.0017 (7)
C7	0.0299 (8)	0.0341 (9)	0.0280 (8)	−0.0069 (7)	−0.0017 (6)	0.0013 (6)
C8	0.0303 (9)	0.0335 (9)	0.0311 (8)	−0.0046 (7)	−0.0011 (6)	−0.0007 (7)
C9	0.0379 (10)	0.0355 (10)	0.0462 (10)	−0.0049 (8)	0.0031 (8)	−0.0008 (8)
C10	0.0544 (12)	0.0365 (10)	0.0526 (12)	0.0058 (9)	0.0034 (10)	−0.0034 (9)
C11	0.0418 (11)	0.0531 (13)	0.0513 (12)	0.0102 (10)	0.0028 (9)	−0.0072 (10)
C12	0.0310 (10)	0.0535 (13)	0.0494 (11)	−0.0034 (9)	0.0057 (8)	0.0002 (9)
C13	0.0315 (9)	0.0409 (10)	0.0389 (9)	−0.0042 (8)	−0.0020 (7)	0.0016 (8)
C14	0.0513 (12)	0.0435 (10)	0.0391 (9)	−0.0056 (10)	0.0053 (8)	−0.0027 (9)
C15	0.0318 (9)	0.0306 (9)	0.0372 (9)	−0.0056 (7)	0.0066 (7)	−0.0007 (7)
C16	0.0406 (10)	0.0294 (8)	0.0391 (9)	−0.0029 (7)	0.0018 (7)	−0.0018 (7)
C17	0.0341 (9)	0.0512 (12)	0.0492 (11)	−0.0046 (9)	0.0052 (8)	−0.0058 (9)
C18	0.0379 (11)	0.089 (2)	0.0546 (13)	−0.0233 (12)	0.0085 (9)	−0.0164 (13)
C19	0.0761 (18)	0.0632 (16)	0.0534 (13)	−0.0401 (15)	0.0107 (12)	−0.0014 (12)
C20	0.0856 (19)	0.0415 (12)	0.0645 (15)	−0.0188 (13)	0.0017 (13)	0.0086 (11)
C21	0.0495 (12)	0.0368 (10)	0.0564 (12)	−0.0059 (9)	−0.0017 (9)	0.0050 (9)
C22	0.0328 (9)	0.0340 (9)	0.0326 (8)	−0.0063 (7)	0.0012 (7)	−0.0034 (7)
C23	0.0263 (8)	0.0258 (8)	0.0396 (9)	0.0011 (6)	−0.0013 (6)	0.0008 (7)
C24	0.0260 (8)	0.0266 (8)	0.0382 (8)	0.0013 (6)	−0.0028 (6)	0.0002 (7)
C25	0.0309 (9)	0.0336 (9)	0.0461 (10)	−0.0024 (8)	0.0034 (7)	−0.0017 (8)
C26	0.0419 (10)	0.0466 (11)	0.0429 (10)	−0.0020 (9)	0.0067 (8)	−0.0078 (8)
C27	0.0472 (11)	0.0480 (12)	0.0473 (11)	−0.0063 (10)	−0.0032 (9)	−0.0140 (9)
C28	0.0404 (10)	0.0452 (11)	0.0520 (11)	−0.0149 (9)	−0.0066 (8)	−0.0033 (9)
C29	0.0307 (9)	0.0357 (9)	0.0398 (9)	−0.0026 (8)	−0.0029 (7)	0.0036 (8)
N1	0.0307 (7)	0.0322 (8)	0.0385 (8)	−0.0061 (6)	0.0031 (6)	0.0001 (6)
N2	0.0345 (7)	0.0323 (7)	0.0376 (7)	−0.0043 (6)	0.0003 (6)	−0.0011 (6)
O1	0.0343 (7)	0.0413 (8)	0.0778 (11)	−0.0062 (6)	0.0092 (7)	0.0116 (8)
O2	0.0440 (8)	0.0613 (10)	0.0410 (7)	−0.0221 (7)	0.0020 (6)	0.0007 (7)

### Geometric parameters (Å, °)

**Table:**

C1—C2	1.380 (3)	C15—H15	0.98
C1—C6	1.385 (3)	C16—N2	1.460 (2)
C1—H1	0.93	C16—H16A	0.97
C2—C3	1.374 (3)	C16—H16B	0.97
C2—H2	0.93	C17—C22	1.385 (3)
C3—C4	1.371 (3)	C17—C18	1.395 (3)
C3—H3	0.93	C17—H17	0.93
C4—C5	1.391 (3)	C18—C19	1.372 (4)
C4—H4	0.93	C18—H18	0.93
C5—C6	1.388 (3)	C19—C20	1.369 (4)
C5—H5	0.93	C19—H19	0.93
C6—C7	1.508 (2)	C20—C21	1.382 (3)
C7—N1	1.283 (2)	C20—H20	0.93
C7—C8	1.469 (3)	C21—C22	1.389 (3)
C8—C9	1.399 (3)	C21—H21	0.93
C8—C13	1.419 (2)	C22—C23	1.500 (2)
C9—C10	1.378 (3)	C23—N2	1.284 (2)
C9—H9	0.93	C23—C24	1.477 (2)
C10—C11	1.388 (3)	C24—C25	1.399 (2)
C10—H10	0.93	C24—C29	1.411 (2)
C11—C12	1.376 (3)	C25—C26	1.383 (3)
C11—H11	0.93	C25—H25	0.93
C12—C13	1.389 (3)	C26—C27	1.386 (3)
C12—H12	0.93	C26—H26	0.93
C13—O1	1.346 (3)	C27—C28	1.376 (3)
C14—C15	1.525 (3)	C27—H27	0.93
C14—H14A	0.96	C28—C29	1.388 (3)
C14—H14B	0.96	C28—H28	0.93
C14—H14C	0.96	C29—O2	1.347 (2)
C15—N1	1.460 (2)	O1—H1A	0.82
C15—C16	1.525 (3)	O2—H2A	0.82
			
C2—C1—C6	120.49 (18)	C16—C15—H15	109.5
C2—C1—H1	119.8	N2—C16—C15	109.55 (15)
C6—C1—H1	119.8	N2—C16—H16A	109.8
C3—C2—C1	120.01 (18)	C15—C16—H16A	109.8
C3—C2—H2	120	N2—C16—H16B	109.8
C1—C2—H2	120	C15—C16—H16B	109.8
C4—C3—C2	120.00 (18)	H16A—C16—H16B	108.2
C4—C3—H3	120	C22—C17—C18	119.5 (2)
C2—C3—H3	120	C22—C17—H17	120.2
C3—C4—C5	120.72 (19)	C18—C17—H17	120.2
C3—C4—H4	119.6	C19—C18—C17	120.0 (2)
C5—C4—H4	119.6	C19—C18—H18	120
C6—C5—C4	119.26 (18)	C17—C18—H18	120
C6—C5—H5	120.4	C20—C19—C18	120.5 (2)
C4—C5—H5	120.4	C20—C19—H19	119.7
C1—C6—C5	119.48 (17)	C18—C19—H19	119.7
C1—C6—C7	118.56 (16)	C19—C20—C21	120.3 (2)
C5—C6—C7	121.90 (16)	C19—C20—H20	119.9
N1—C7—C8	119.60 (16)	C21—C20—H20	119.9
N1—C7—C6	122.34 (17)	C20—C21—C22	119.8 (2)
C8—C7—C6	118.02 (15)	C20—C21—H21	120.1
C9—C8—C13	117.66 (17)	C22—C21—H21	120.1
C9—C8—C7	121.20 (17)	C17—C22—C21	119.81 (18)
C13—C8—C7	121.13 (15)	C17—C22—C23	121.07 (18)
C10—C9—C8	122.01 (19)	C21—C22—C23	119.12 (16)
C10—C9—H9	119	N2—C23—C24	118.18 (15)
C8—C9—H9	119	N2—C23—C22	123.25 (16)
C9—C10—C11	119.3 (2)	C24—C23—C22	118.55 (15)
C9—C10—H10	120.4	C25—C24—C29	117.87 (16)
C11—C10—H10	120.4	C25—C24—C23	121.07 (15)
C12—C11—C10	120.5 (2)	C29—C24—C23	121.06 (15)
C12—C11—H11	119.8	C26—C25—C24	121.64 (18)
C10—C11—H11	119.8	C26—C25—H25	119.2
C11—C12—C13	120.7 (2)	C24—C25—H25	119.2
C11—C12—H12	119.6	C25—C26—C27	119.29 (19)
C13—C12—H12	119.6	C25—C26—H26	120.4
O1—C13—C12	118.80 (18)	C27—C26—H26	120.4
O1—C13—C8	121.39 (17)	C28—C27—C26	120.58 (19)
C12—C13—C8	119.81 (18)	C28—C27—H27	119.7
C15—C14—H14A	109.5	C26—C27—H27	119.7
C15—C14—H14B	109.5	C27—C28—C29	120.45 (19)
H14A—C14—H14B	109.5	C27—C28—H28	119.8
C15—C14—H14C	109.5	C29—C28—H28	119.8
H14A—C14—H14C	109.5	O2—C29—C28	117.95 (17)
H14B—C14—H14C	109.5	O2—C29—C24	121.87 (16)
N1—C15—C14	108.39 (15)	C28—C29—C24	120.17 (17)
N1—C15—C16	109.14 (14)	C7—N1—C15	122.13 (16)
C14—C15—C16	110.66 (15)	C23—N2—C16	121.53 (16)
N1—C15—H15	109.5	C13—O1—H1A	109.5
C14—C15—H15	109.5	C29—O2—H2A	109.5
			
C6—C1—C2—C3	2.0 (3)	C19—C20—C21—C22	−0.1 (4)
C1—C2—C3—C4	−0.7 (3)	C18—C17—C22—C21	0.4 (3)
C2—C3—C4—C5	−0.5 (4)	C18—C17—C22—C23	179.17 (18)
C3—C4—C5—C6	0.5 (4)	C20—C21—C22—C17	−0.2 (3)
C2—C1—C6—C5	−1.9 (3)	C20—C21—C22—C23	−179.00 (19)
C2—C1—C6—C7	175.34 (18)	C17—C22—C23—N2	−93.9 (2)
C4—C5—C6—C1	0.7 (3)	C21—C22—C23—N2	84.9 (2)
C4—C5—C6—C7	−176.5 (2)	C17—C22—C23—C24	87.8 (2)
C1—C6—C7—N1	−87.8 (2)	C21—C22—C23—C24	−93.4 (2)
C5—C6—C7—N1	89.5 (2)	N2—C23—C24—C25	172.54 (17)
C1—C6—C7—C8	89.8 (2)	C22—C23—C24—C25	−9.1 (2)
C5—C6—C7—C8	−92.9 (2)	N2—C23—C24—C29	−7.8 (2)
N1—C7—C8—C9	−171.73 (18)	C22—C23—C24—C29	170.61 (16)
C6—C7—C8—C9	10.6 (2)	C29—C24—C25—C26	0.5 (3)
N1—C7—C8—C13	7.3 (3)	C23—C24—C25—C26	−179.84 (18)
C6—C7—C8—C13	−170.35 (16)	C24—C25—C26—C27	0.3 (3)
C13—C8—C9—C10	0.1 (3)	C25—C26—C27—C28	−0.9 (3)
C7—C8—C9—C10	179.19 (17)	C26—C27—C28—C29	0.7 (3)
C8—C9—C10—C11	1.1 (3)	C27—C28—C29—O2	−178.9 (2)
C9—C10—C11—C12	−0.7 (3)	C27—C28—C29—C24	0.2 (3)
C10—C11—C12—C13	−0.8 (3)	C25—C24—C29—O2	178.35 (17)
C11—C12—C13—O1	−178.8 (2)	C23—C24—C29—O2	−1.4 (3)
C11—C12—C13—C8	2.0 (3)	C25—C24—C29—C28	−0.7 (3)
C9—C8—C13—O1	179.18 (19)	C23—C24—C29—C28	179.59 (17)
C7—C8—C13—O1	0.1 (3)	C8—C7—N1—C15	177.40 (14)
C9—C8—C13—C12	−1.7 (3)	C6—C7—N1—C15	−5.0 (3)
C7—C8—C13—C12	179.27 (17)	C14—C15—N1—C7	−111.3 (2)
N1—C15—C16—N2	−66.08 (18)	C16—C15—N1—C7	128.14 (18)
C14—C15—C16—N2	174.74 (15)	C24—C23—N2—C16	−175.93 (15)
C22—C17—C18—C19	−0.2 (3)	C22—C23—N2—C16	5.8 (3)
C17—C18—C19—C20	−0.1 (4)	C15—C16—N2—C23	131.47 (17)
C18—C19—C20—C21	0.3 (4)		

### Hydrogen-bond geometry (Å, °)

**Table:**

*D*—H···*A*	*D*—H	H···*A*	*D*···*A*	*D*—H···*A*
O1—H1A···N1	0.82	1.84	2.573 (2)	147
O2—H2A···N2	0.82	1.83	2.553 (2)	147
